# Epidemiology and Characteristics of Cervical Spine Injury in Patients Presenting to a Regional Emergency Department

**DOI:** 10.7759/cureus.2179

**Published:** 2018-02-10

**Authors:** Etimbuk Umana, Khalid Khan, MN Baig, James Binchy

**Affiliations:** 1 Department of Emergency Medicine, University Hospital Galway; 2 Trauma & Orthopaedics, University Hospital Galway

**Keywords:** cervical spine injury, incidence, computer tomography

## Abstract

Purpose

This study aims to establish the demographics and characteristics of patients with cervical spine injury (CSI) in an Irish cohort presenting to a regional emergency department.

Methods

We performed a retrospective analysis of the medical records of patients who underwent cervical spine computed tomography (CT) scans following trauma from January 2013 to July 2016. We looked at the mechanism of injury, mode of arrival to the emergency department, triage category, correlation between examination, and site of CSI and neurological status.

Results

Over the study period, 808 patients underwent CT scans of the cervical spine for potential CSI. The incidence of CSI in our cohort was 9.4% (n = 76). Approximately 70% (n = 53) were men. Falls (53%) and motor vehicle accidents (29%) were noted to be the more common mechanisms of injury in this cohort. The C2 region was the most common location for CSI. Only 7% (n = 5) of patients had documented neurology.

Conclusion

This study demonstrates the epidemiology and characteristics of CSI presenting in an Irish cohort. The incidence of CSI was found to be 9.4% with a male preponderance and falls being the most common cause of trauma.

## Introduction

Cervical spine injury (CSI) has been widely reported in patients with blunt trauma and represents an important subgroup presenting to the Emergency Department (ED). There is a variation in incidence and epidemiological data of patients presenting with CSI in different populations [1-7]. The incidence of CSI ranges from 2% to 12%, with a higher incidence in those who are obtunded or intoxicated and patients who are difficult to evaluate clinically [1-4]. Most studies have reported a bimodal age distribution with the first peak between 15 years to 45 years and a second peak for those aged 65 years to 80 years [1,2,8,9]. With respect to gender, CSI is more common in men [1,2,5-7]. CSI with cord injury can be devastating and can lead to permanent, irreversible disability. Therefore, patients with suspected CSI undergo cervical spine immobilization and systematic evaluation to identify patients with significant injuries. This study aims to establish the demographics and characteristics of patients with CSI presenting to an Irish regional ED. These characteristics include mechanism of injury, mode of arrival to the ED, triage category, the correlation between examination and site of CSI, neurological status, and management outcome.

## Materials and methods

This was a retrospective study carried out in a regional tertiary university hospital in the West of Ireland with an annual attendance of over 65,000 presentations to the ED. This study period was from January 2013 to July 2016, inclusive. The patients who underwent cervical spine computed tomography (CT) scan following blunt trauma to the cervical spine were identified through the hospital radiology registry (PACS System). Variables retrieved included age, gender, triage category, Glasgow Coma Scale (GCS), mechanism and site of injury, mode of arrival, and clinical correlation with fracture site on CT. Neurological status and management outcomes were also documented.

## Results

**Figure 1:**
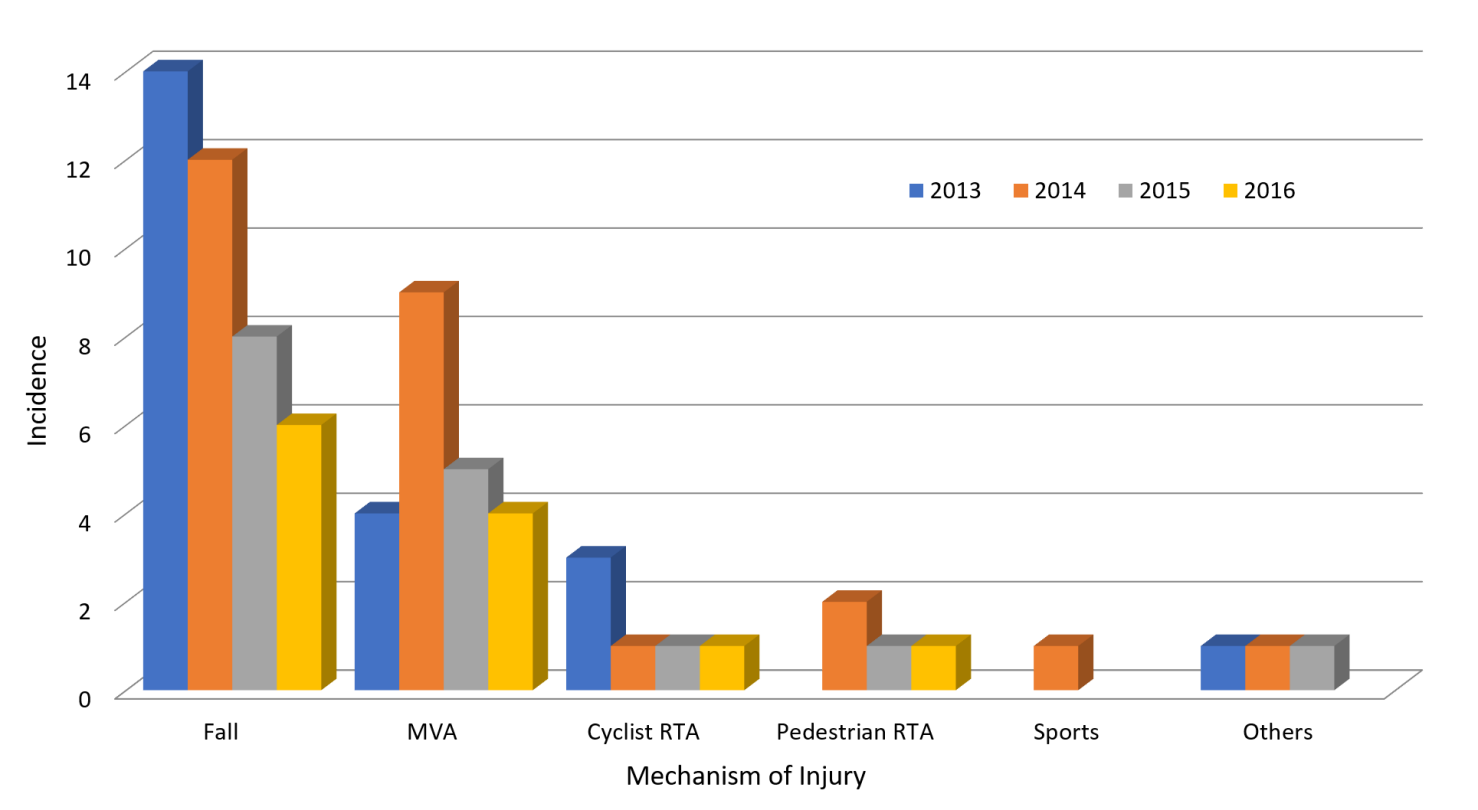
Trends of mechanism of injury in cervical spine injuries. MVA: Motor vehicle accident; RTA: Road traffic accident.

Over the study period, 808 patients underwent CT cervical spine for potential CSI. The incidence of CSI in our cohort was 9.4% (n = 76). Approximately 70% (n = 53) were men, and the mean age of patients presenting with CSI was 53.8 (standard deviation (SD), 23.2). Approximately 61% of patients were under 60 years of age but only 7.9% were under 20 years of age. Table 1 gives an overview of patient characteristics presenting with CSI. Falls (53%) and motor vehicle accidents (MVA; 29%) were the more common mechanisms of injury in our patient cohort. Cyclist road traffic accident (RTA; 8%), pedestrian RTA (5%), sports, (1%) and others (4%) made up the remainder. A decreasing trend regarding the incidence of falls was noted from 2013 to 2016 (Figure 1).

**Table 1:** Patient characteristics (n = 76). SD: Standard deviation; MVA: Motor vehicle accident; RTA: Road traffic accident; MTS: Manchester Triage System; N/A: Not assessed.

Characteristics		% (n)
Gender	Male	70% (53)
	Female	30% (23)
Age	Mean/SD (Range)	53.8/23.2 (6—99)
Mechanism of injury	Falls	53% (40)
	MVA	29% (22)
	Cyclist RTA	8% (6)
	Pedestrian RTA	5% (4)
	Sports	1% (1)
	Others	4% (3)
Mode of arrival	Ambulance	76.3% (58)
	Helicopter	2.6% (2)
	Private transport	21.1% (16)
Alcohol related		9% (7)
GCS	Mean/SD (Range)	13.9/2.7 (3—15)
MTS	Category 1	21% (16)
	Category 2	43% (33)
	Category 3	28% (21)
	Category 4	8% (6)
Neurology	Abnormal	7% (5)
	Intact	85% (65)
	N/A	8% (6)
Management	Conservative	76% (58)
	Surgery	24% (18)

 

**Figure 2:**
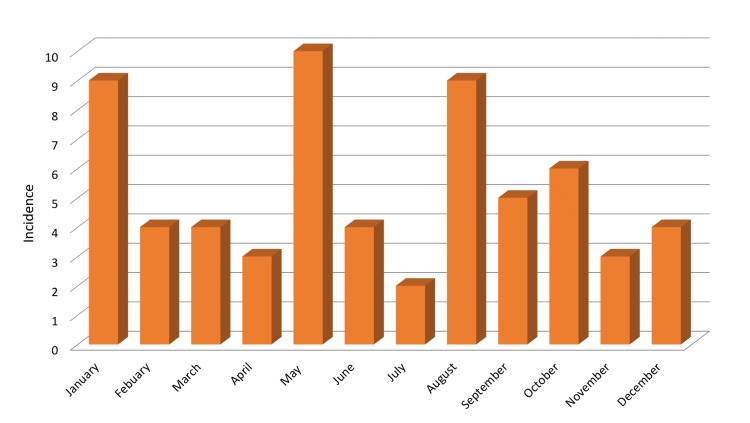
Seasonal trend in injury presentation.

**Figure 3:**
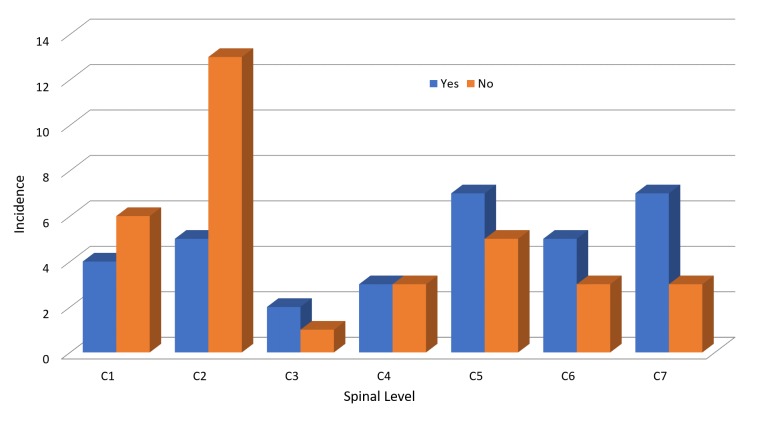
Clinical correlation with fracture site.

Most patients arrived at the ED by ambulance (76%). An assessment of the seasonal trend of injury presentations revealed that January, May, and August are the more common months for injury presentation (Figure 2). Only 9% (n = 7) of patients with CSI were noted to have an alcohol-related presentation. The mean GCS for our cohort was 13.9 (SD, 2.7), with some patients presenting with a GCS as low as 3. Patients were triaged according to the Manchester Triage System and were given one of four triage categories. Most patients were triaged as Category 1 or 2 (64.5%), while 35.5% were triaged Category 3 or 4. On examination, 67 patients had a documented area of tenderness. Of the 67 patients, a clinical exam correlated with the site of the fracture on the cervical spine CT scan in 49% (n = 33) of patients. Figure 3 illustrates the correlation between clinical exam and fracture site.

 

**Figure 4:**
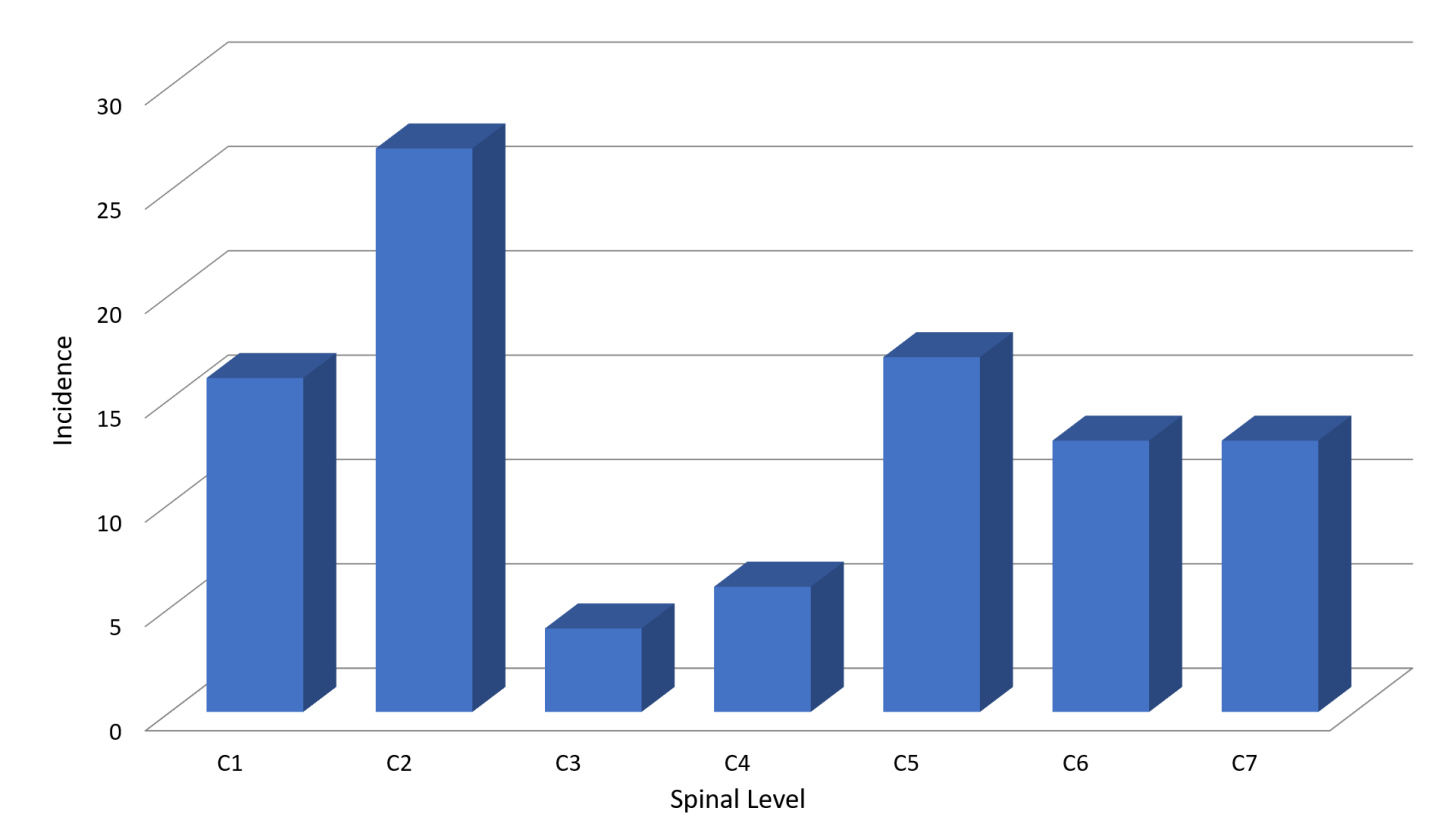
Level of injury.

The C2 region was the most common level for the location of CSI (Figure 4), closely followed by C5 and C1 regions. The highest incidence of C2 fractures was observed in those above 80 years of age with falls representing the most common mechanism of injury. Sixteen patients had more than one level of CSI, with C1-C2, C5-C6, and C5-C6-C7 representing the more common combination of injury. Only 7% (n = 5) of patients had documented abnormal neurology, the rest either had intact neurology or no documentation of neurological status. Seventy-six percent (n = 58) of the patients with CSI were managed conservatively while the rest needed surgical intervention.

## Discussion

Our study will be the first to focus on patients presenting with CSI from the general population in the West of Ireland. The incidence of CSI in our cohort was 9.4%, which is within the range of 2% to 12% found in the published literature [1-5]. Unlike past studies that described a bimodal age distribution in patients presenting with CSI [1,2,8-10], we found that 61% of patients were younger than 60 years of age, with a peak incidence between 20 and 30 years and 50 and 60 years of age. The remaining 39% included those older than 60 years with a peak incidence occurring between the ages of 80 and 90 years of age. Other studies have reported an increased incidence of CSI with increasing age, a trend we did not observe in our study [2,6]. In terms of gender distribution, our study found a male predominance of 70%, which aligns with previous studies on CSI [1,2,5-7].

Very few authors report falls as the most common mechanism of injury for CSI [6], with the majority reporting MVAs as the most common followed closely by falls [1,2,4,7]. We also noted the mean age for falls was 61.1 years which was higher than other causes of injury in our study. The decreasing incidence of falls in the data set could be associated with measures to increase safety at work and fall prevention programs instituted within the community. Urdaneta, et al. analyzed patients with CSI based on the mode of arrival and found 93% of patients arrived via Emergency Medical Services while 7% arrived via private transport. Twenty-one percent of our study population arrived by private transport and were more likely to be triaged as Category 3 and 4, with two patients requiring surgical intervention. As Urdaneta, et al. stipulated, patients arriving by private transport represent a low-risk group with potential for significant injury requiring surgery, and ED providers should maintain high levels of vigilance for CSI in this cohort. C2 was the most common level involved for CSI, which aligns with prior studies [5,6]. This was closely followed by C5 and C1 cervical vertebrae. Though injury at C2 was the most common level of CSI reported, clinical exams did not correlate with the fracture site on cervical spine CT scans. In comparison, tenderness over C5 to C7 on examination was more likely to correlate with a fracture site on a cervical spine CT.

Understanding the demographics of our patient population, their mechanism of injury, and expected findings can better help us prepare to manage this cohort when they present to the ED.

## Conclusions

This study demonstrates the epidemiology and characteristic of CSI presenting in an Irish cohort. The incidence of CSI was found to be 9.4% with a male preponderance and falls being the most common cause of trauma. This study is important as it would help with resource and personnel allocation to improve our services and optimize the management of patients presenting with CSI.

## References

[1] Clayton JL, Harris MB, Weintraub SL (2012). Risk factors for cervical spine injury. Injury.

[2] Lowery DW, Wald MM, Browne BJ (2001). Epidemiology of cervical spine injury victims. Ann Emerg Med.

[3] MacDonald RL, Schwartz ML, Mirich D (1990). Diagnosis of cervical spine injury in motor vehicle crash victims: how many X-rays are enough?. J Trauma.

[4] Martin MJ, Bush LD, Inaba K (2017). Cervical spine evaluation and clearance in the intoxicated patient: a prospective Western Trauma Association Multi-Institutional Trial and Survey. J Trauma Acute Care Surg.

[5] Malik SA, Murphy M, Connolly P (2008). Evaluation of morbidity, mortality and outcome following cervical spine injuries in elderly patients. Eur Spine J.

[6] Fredø HL, Rizvi SA, Lied B (2012). The epidemiology of traumatic cervical spine fractures: a prospective population study from Norway. Scand J Trauma Resusc Emerg Med.

[7] Yadollahi M, Paydar S, Ghaem H (2016). Epidemiology of cervical spine fractures. Trauma Mon.

[8] McCabe JB, Angelos MG (1984). Injury to the head and face in patients with cervical spine injury. Am J Emerg Med.

[9] Ryan MD, Henderson JJ (1992). The epidemiology of fractures and fracture-dislocations of the cervical spine. Injury.

[10] Baig M (2017). A review of epidemiological distribution of different types of fractures in paediatric age. Cureus.

